# Contrasting Range Shifts of an Endangered Orchid *Changnienia amoena* and Its Obligate Pollinator Under Climate Change in China

**DOI:** 10.3390/biology15060485

**Published:** 2026-03-19

**Authors:** Yue Wang, Songwen Guo, Jingxin Zhou, Guangfu Zhang

**Affiliations:** Jiangsu Key Laboratory of Biodiversity and Biotechnology, School of Life Sciences, Nanjing Normal University, Wenyuan Road, Nanjing 210023, China; 09240205@njnu.edu.cn (Y.W.); 09240337@njnu.edu.cn (S.G.); 09230132@njnu.edu.cn (J.Z.)

**Keywords:** Biomod2, conservation, environmental factors, Orchidaceae, population centroid, suitable range

## Abstract

Specialized plant–pollinator interactions are highly vulnerable to climate change, yet predicting their coupled responses remains challenging. The endangered orchid *Changnienia amoena* and its sole effective pollinator, *Bombus trifasciatus*, represent a great system to study such dynamics in China. However, existing research on their potential distributions shows substantial discrepancies due to methodological variations. We applied ensemble modeling in Biomod2, integrating multiple algorithms, to project the current and future (2041–2100, under SSP1-2.6, SSP2-4.5, and SSP5-8.5 scenarios) distributions of both species. Models were built using comprehensive occurrence records (123 for *C. amoena*, 43 for *B. trifasciatus*) and key environmental variables. Annual precipitation primarily affected the distribution of *C. amoena*, while precipitation of the driest quarter was key for *B. trifasciatus*. Under climate change, *C. amoena* is projected to contract slightly in suitable habitat and shift southeastward, whereas *B. trifasciatus* may expand in climatic suitability. Niche overlap between the two species is predicted to decrease, indicating a potential future spatial mismatch. This study is the first to predict the current suitable habitat and future changes in the endangered orchid and its pollinator through ensemble modeling. As a result, it highlights contrasting range shifts in this specialized mutualism, underscoring the risk of climate-driven interaction disruption. The findings provide a critical scientific basis for developing targeted conservation strategies for *C. amoena* that consider the spatial ecology of its obligate pollinator.

## Simple Summary

Climate change threatens the survival of the endangered Chinese orchid *Changnienia amoena*, which is pollinated mainly by the bumblebee *Bombus trifasciatus*. Our study predicts where both species live now and where they will live in the future. As temperatures rise, the orchid’s suitable habitat may shrink and shift southeast, while its pollinator shows a westward shift in climatic suitability. This indicates the two species are likely to move in opposite directions, reducing their chances of meeting. Such a mismatch may lower pollination efficiency, thereby reducing the orchid’s distribution. These findings highlight the need for conservation plans that safeguard both the orchid and its obligate bee together, ensuring they continue to coexist.

## 1. Introduction

Amidst ongoing global warming and increasing occurrences of extreme weather events, significant shifts are being observed in species distributions, phenology, behavior, and adaptive capacity [1]. These changes pose considerable challenges to population dynamics and genetic diversity, especially for endemic plant species with restricted ranges [2,3]. Climate change reduces ecosystem resilience to fluctuations in temperature and precipitation, elevates risks of plant water stress, results in habitat fragmentation and degradation, and even prompts species shift toward higher latitudes or northern regions [4,5,6]. Orchid species are particularly vulnerable to climatic shifts owing to traits such as mycorrhizal specificity, specialized pollination systems, and low seed germination rates [7,8]. For instance, under combined pressures from climate and land cover change, suitable habitats for both lowland and upland orchids endemic to New Guinea are projected to decline, with both groups exhibiting upward elevational shifts under future climate scenarios [9]. Similarly, a study of 17 endangered orchid species on the Qinghai–Tibet Plateau reveals that species richness decreases with increasing elevation in China and that topographic (slope, aspect, elevation) and climatic (precipitation, temperature) factors significantly influence the potential distribution of Orchidaceae across the region [10]. Additionally, among angiosperms in China, the family with the highest number of threatened species is Orchidaceae, comprising 653 species, which accounts for 19.42% of all Chinese threatened angiosperms and 43.48% of the total species within the Orchidaceae family [11].

Likewise, climate change exerts profound impacts on pollinator insects besides orchids. Global warming increases the likelihood of extreme weather events, such as heatwaves and droughts, which may exceed the physiological tolerance thresholds for many insect species. These extremes can directly affect survival and development through thermal stress or indirectly disrupt insect life cycles by altering host plant resource availability, leading to shifts in instar number, developmental timing, or the induction of diapause [12]. As a key functional group in ecosystems, the community composition and functioning of pollinator insects are strongly influenced by climatic conditions [13]. Among them, obligatory pollinators are particularly vulnerable due to their narrow ecological niches and high dependence on specific hosts [14].

Moreover, climate change can induce phenological mismatches between plant flowering and pollinator activity [15], thereby reducing pollination effectiveness. Such asynchrony tends to affect specialized pollination systems more severely than generalized ones [16]. As one of the most species-rich families of seed plants worldwide, Orchidaceae includes approximately one-third of species that are estimated to employ deceptive pollination strategies [17,18,19]. Among these, sexually deceptive orchids exhibit extremely high pollination specificity, often relying on a single pollinator species, which renders their mutualistic interactions especially sensitive to climatic variability [17]. Consequently, climate change may threaten not only orchid populations and their specific pollinators but also the stability of the highly specialized interaction networks that sustain them.

Nonetheless, it remains unclear how climate change affects the interactive systems between orchids and their specialized pollinators. For instance, in studies of sexually deceptive orchids, some researchers have attempted to integrate pollinator data into Species Distribution Models (SDMs). By quantifying and comparing predictions with and without pollinator information, these studies underscore the ecological importance of plant–pollinator interactions [13,20]. It should be noted, however, that such work has often relied on a single algorithm, such as MaxEnt. Compared with ensemble modeling that combines multiple algorithms, results from a single model may be less robust and accurate. Kolanowska and Michalska [21] examined spatial divergence in suitable habitat between the Australian endemic orchid *Cryptostylis leptochila* F. Muell. and its specific pollinator *Lissopimpia excelsa* Costa under future climate scenarios. However, they only selected bioclimatic variables, excluding topographic and anthropogenic factors, and they used a fixed threshold for binary habitat classification, which may obscure suitability gradients and incur bias in distribution projections. These studies analyzed the response of specialized orchid-pollinator interaction systems to climate change, shifting the analytical perspective from single species to species interactions using MaxEnt modeling. In fact, they also reveal current challenges in model algorithms, variable selection, and habitat suitability classification. Among the many such interactive systems, the specific relationship between *Changnienia amoena* S. S. Chien and *Bombus trifasciatus* (Smith, 1852) has been considered a typical model for investigating climate-driven changes in specialized plant–pollinator interactions due to its well-documented specificity [22].

*C. amoena* is a rare and endangered orchid endemic to China. It was listed as a second-grade species in the List of National Key Protected Wild Plants approved by the State Council in 2021 and is also assessed as an “Endangered” (EN) species on the IUCN Red List [23]. This orchid faces multiple survival threats: firstly, it employs a unique deceptive pollination strategy, offering no nectar reward to its pollinators, resulting in extremely low seed set rates in the wild [24]; secondly, it is difficult for its wild populations to regenerate, together with an increasingly patchy distribution and severely fragmented habitats in China [25]; thirdly, due to its high ornamental and medicinal value, it suffers from over-exploitation and illegal collection. Furthermore, *C. amoena* has an ancient origin and retains numerous ancestral traits and, accordingly, can serve as important material for the systematic phylogeny of Orchidaceae [26]. *B. trifasciatus* is currently the only known effective pollinator of *C. amoena* in the wild [22,27]. It possesses an elongated mouthpart that is morphologically adapted to the floral tube of *C. amoena*, and thereby its distribution directly affects the seed-setting rate and population persistence of this orchid [27]. Notably, *B. trifasciatus* is a generalist pollinator that utilizes a broad range of flowering plant resources. As a result, its distribution is minimally affected by a reduction in niche overlap, and the loss of any single plant species does not threaten its overall population survival [28].

Species Distribution Models (SDMs) are important tools that use species distribution data and environmental variables to predict the potential geographical distribution of species through algorithms. They are now widely applied in studies on species’ geographic distribution and conservation of endangered species [29]. In recent years, researchers have employed the Biomod2 platform to establish an ensemble model. This platform integrates ten single models, such as ANN (Artificial Neural Networks), MAXENT (Maximum Entropy), RF (Random Forest), etc. By leveraging the strengths of multiple models, the ensemble model can effectively reduce the uncertainty of single models and significantly improve prediction reliability [30,31].

Research on the potential distribution of *C. amoena* and its pollinating insects in China remains limited and inconsistent. Although Liu et al. [22] suggested that future habitats for both species may expand, no consensus exists on the current suitable habitats of *C. amoena*. Estimates of its suitable area vary considerably across studies: Liu et al. [23] reported 58.33 × 10^4^ km^2^ using 93 occurrence points and Biomod2; Liu et al. [32] obtained 16.47 × 10^4^ km^2^ with 48 points and MaxEnt; while Liu et al. [22] integrated 69 points of *C. amoena* and 34 of *B. trifasciatus* using MaxEnt and GTWR, yielding 43.56 × 10^5^ km^2^ for *C. amoena*. Moreover, key environmental drivers identified differ among these studies. Such discrepancies likely stem from differences in sample representativeness and coverage [33], modeling algorithms, habitat delineation criteria, and variable selection. Specifically, Liu et al. [22] employed the natural break method for habitat classification, which may be unsuitable for rare and endangered species like *C. amoena*; the MaxSSS threshold is considered more appropriate when only presence data are available [34], particularly for threatened plants [35,36]. Additionally, their sample size (69) was smaller than that of Liu et al. [23] (93), and their distribution map omitted records from Henan Province documented in previous studies [23,37,38]. Therefore, the response of *C. amoena* and its pollinator to climate change requires further investigation using more comprehensive data and robust methods. Here, based on the ensemble modeling from the Biomod2 platform, we evaluate the habitat suitability of the endangered *C. amoena* and its pollinator under climate change. More specifically, we will answer the following questions: (1) What are the key environmental factors influencing the distribution of *C. amoena* and its pollinator, respectively? (2) How will climate change alter the potential suitable habitats for *C. amoena* and *B. trifasciatus* under current and future scenarios? (3) Whether a “mismatch” between them will occur in their suitable habitats? The study aims to reveal the potential distributional impacts of climate change on the specialized interaction system between *C. amoena* and its pollinator, providing a scientific reference for future conservation and management strategies for *C. amoena* and other endangered orchids in China.

## 2. Materials and Methods

### 2.1. Species Occurrence Records

In this study, distribution records for the endangered orchid *C. amoena* and its pollinator *B. trifasciatus* were compiled from three sources: field surveys, online databases, and a systematic literature review.

Field surveys were conducted from 2021 to 2025 mainly in the eastern Chinese provinces like Anhui, Jiangsu, Jiangxi, and Zhejiang. These surveys resulted in the discovery of several new populations, which were integrated into the distribution dataset.

Online databases were queried to obtain additional occurrence data. This included: (1) specimen records with precise coordinates or detailed locality information from the National Specimen Information Infrastructure of China (NSII, http://www.nsii.org.cn, accessed on 12 November 2025) and the Global Biodiversity Information Facility (GBIF, https://www.gbif.org/, accessed on 12 November 2025); and (2) georeferenced images from the Plant Photo Bank of China (PPBC, http://ppbc.iplant.cn, accessed on 10 November 2025).

The literature review involved systematically consulting the Flora of China, regional floras, published papers, and survey reports [22,23] to supplement the specimen and survey data.

For records with only textual descriptions lacking coordinates, Google Earth was used for georeferencing, with latitude and longitude retained to two decimal places. All records were carefully screened; duplicates, cultivated individuals, and those with ambiguous or unverifiable locations were excluded.

**Figure 1:**
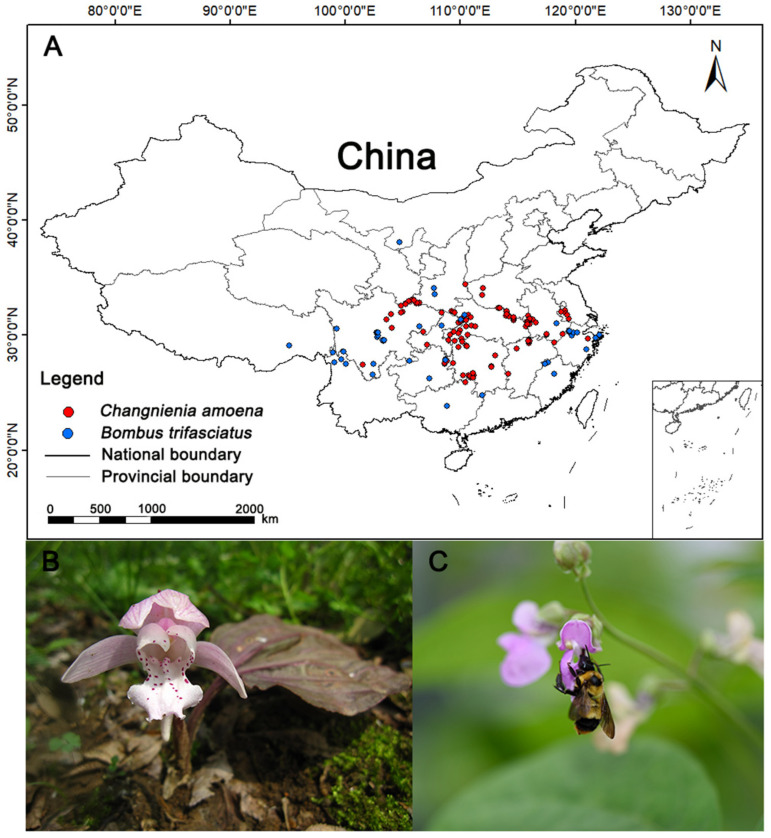
(**A**) Occurrence records of the orchid *Changnienia amoena* and its pollinator *Bombus trifasciatus* in China; (**B**) *Changnienia amoena*—flower (Photographed by Guangfu Zhang); (**C**) *Bombus trifasciatus*—adult (Photographed by Jiaxing Huang).

Initially, 125 occurrence points for *C. amoena* and 48 for *B. trifasciatus* were collected. To reduce sampling bias and spatial autocorrelation, the “Spatially Sparse Occurrence Data for SDMs” tool in SDMtoolbox 2.0 was applied with a spatial resolution of 1 km [39,40]. After processing, 123 records for *C. amoena* and 43 for *B. trifasciatus* were retained for modeling (Figure 1; Supplementary Table S1).

### 2.2. Environmental Variables

Species distributions are often strongly influenced by climatic conditions [41]. Field observations and previous studies indicate that *C. amoena* primarily occurs in moist secondary forests in subtropical mountainous regions of central and eastern China [24]. Its primary pollinator, *B. trifasciatus*, occurs mainly in low- to mid-elevation mountain landscapes in eastern and southwestern China, typically in semi-natural habitats subject to frequent human disturbance [24,27]. Given these ecological characteristics, we selected three categories of predictors:

(1) Climatic variables: 19 bioclimatic variables were obtained from WorldClim for current and future periods (i.e., 2041–2060, 2061–2080, 2081–2100). Current climate data were sourced from WorldClim version 2.1 at 30 arc-second resolution (~1 km), while future climate projections were based on the Beijing Climate Center Climate System Model version 2 with Medium Resolution (BCC-CSM2-MR) global climate model (CMIP6), which performs well in Asia, especially China [42,43]. Three Shared Socioeconomic Pathways (SSP1-2.6, SSP2-4.5, and SSP5-8.5) were selected, representing optimistic, moderate, and pessimistic scenarios, respectively-.
(2) Topographic variables: Elevation and aspect data were obtained from WorldClim (https://www.worldclim.org/, accessed on 15 November 2025), and slope was derived from a Digital Elevation Model (DEM) (http://www.tuxingis.com, accessed on 15 November 2025).
(3) Anthropogenic variables: The global Human Influence Index dataset from NASA’s Socioeconomic Data and Applications Center (SEDAC) (https://sedac.ciesin.columbia.edu, sources accessed on 15 November 2025) was used. The Human Influence index is a composite metric derived from nine integrated data layers: population density, built-up areas, nighttime lights, land use/cover, and transportation networks (roads, railways, coastlines, and navigable rivers) [44].

A total of 23 environmental variables (i.e., climatic, topographic, and anthropogenic) were initially considered (Table 1). To avoid overfitting, the “Remove Highly Correlated Variables” tool in SDMtoolbox 2.0 was applied with a maximum correlation threshold of 0.8. When the absolute Pearson correlation coefficient between two variables exceeded 0.8, the variable with the higher contribution was retained. Finally, 9 variables were selected for *C. amoena* (i.e., 5 climatic, 3 topographic, 1 anthropogenic) and 9 for *B. trifasciatus* (i.e., 5 climatic, 3 topographic, 1 anthropogenic).

### 2.3. Modeling Process

We used the Biomod2 package (version 3.5.1) to simulate the potential distributions of *C. amoena* and its pollinator under current climate conditions. First, ten individual models available on the Biomod2 platform were run. During modeling, 1000 pseudo-absence points were randomly generated using R 4.4.1, and occurrence data were randomly split into 75% for training and 25% for validation. For each model, the Area Under the Curve (AUC) and True Skill Statistic (TSS) were calculated based on the validation set, and these metrics served as the criteria for model selection [45]. This procedure was repeated 10 times, with the mean prediction used as the final model output, and the corresponding AUC and TSS values were obtained.

AUC values indicate model predictive accuracy, with higher values denoting both stronger model-environment correlation and greater predictive reliability. AUC is typically classified as excellent (>0.9), good (0.8–0.9), fair (0.7–0.8), and poor (0.6–0.7) [46]. TSS ranges from −1 to 1. Based on established criteria, models with TSS > 0.8 are considered excellent; scores of 0.6–0.8, 0.4–0.6, and 0.2–0.4 denote good, fair, and poor performance, respectively, while values below 0.2 indicate a failed model [47].

Following published benchmarks, we retained ensemble models that met predefined performance thresholds: for *C. amoena*, AUC > 0.90 and TSS > 0.70 [48,49]; for *B. trifasciatus*, AUC > 0.8 and TSS > 0.7 [50,51]. Models with AUC and TSS values of 1 were excluded to avoid overfitting. Selected models were used to predict potentially suitable habitats under current and nine future climate scenarios.

In presence–pseudoabsence SDMs, a perfect discrimination score (AUC = 1 and TSS = 1) can indicate that the model has captured idiosyncrasies of the pseudoabsence draw and/or the training–validation split rather than generalizable environmental constraints. Such a “perfect” model may therefore have limited transferability and can lead to overconfident spatial projections. For this reason, we treated models with AUC = 1 and TSS = 1 as overfitted and excluded them from the final ensemble model used for projection [52,53].

### 2.4. Geospatial Data Analysis

To delineate potential distribution changes in *C. amoena* and its pollinator under different climate scenarios, ensemble model results were mapped using ArcMap 10.8. Based on the “maximum sum of sensitivity and specificity” criterion [34], the Maximum Sum of Sensitivity and Specificity thresholds (MaxSSS thresholds) were set to 0.1664 for *C. amoena* and 0.2242 for *B. trifasciatus*. Habitat suitability for *C. amoena* was classified as: unsuitable (0.00–0.17), low (0.17–0.44), medium (0.44–0.72), and high (0.72–1.00). For *B. trifasciatus*, it was classified as: unsuitable (0.00–0.22), low (0.22–0.48), medium (0.48–0.74), and high (0.74–1.00).

For the plant (*C. amoena*), total suitable area was defined as the sum of medium and high suitability areas [54,55]. For the insect (*B. trifasciatus*), total suitable area included low, medium, and high suitability areas [31,56]. Furthermore, centroid shifts were calculated using SDM Toolbox v2.5 to assess the impact of climate change on species distributions.

### 2.5. Niche Overlap Metrics

The degree of niche overlap serves as a measure of ecological similarity between species, reflecting their congruence in resource utilization and habitat selection [57]. We used ENMTools v1.3.1 to calculate Schoener’s *D* and Hellinger’s *I* to quantify the niche overlap between *C. amoena* and *B. trifasciatus*. The formula for *D* is [58]:

$$D\left(p_{X},\;p_{Y}\right)=1-\frac{1}{2}\sum_{i}|p_{X,i}-p_{Y,i}|$$

The *D* index is computed by comparing the suitability values of species *X* and *Y* in each grid cell to assess distributional similarity; it ranges from 0 to 1, where 0 indicates no overlap and 1 indicates complete overlap [59]. *D* values are typically categorized into the following levels: extremely high overlap (0.80–1.00), high overlap (0.60–0.80), moderate overlap (0.40–0.60), low overlap (0.20–0.40), and no or very low overlap (0.00–0.20) [36,60]. The formula for calculating the *I* index is:

$$I\left(p_{X},\;p_{Y}\right)=\frac{1}{2}\left.\sqrt{\sum_{i}{\left(\sqrt{p_{X,i}}-\sqrt{p_{Y,i}}\right)}^{2}}\right.$$

*I* also ranges from 0 to 1, where 0 indicates no overlap and 1 indicates identical environmental requirements [58,61].

## 3. Results

### 3.1. Model Performance

Using Biomod2, ten individual models were built for *C. amoena* and *B. trifasciatus*. The final datasets consisted of 123 records and 9 environmental variables for *C. amoena* and 43 records and 9 variables for *B. trifasciatus*. To evaluate the accuracy of the models, we computed the AUC and TSS values for each individual model as well as for the ensemble model (Table 2). We then applied an optimal ensemble modeling algorithm to screen and select the models: For *C. amoena*, only models with AUC > 0.9 and TSS > 0.7 were retained; ANN and SRE were excluded. For *B. trifasciatus*, models with AUC > 0.8 and TSS > 0.7 were retained; ANN, SRE, CTA, and FDA were excluded. The RF model exhibited AUC and TSS values of 1.000, indicating clear signs of overfitting, and was therefore excluded from the construction of the species-pair ensemble model. Ultimately, we retained 7 models for *C. amoena* and 5 for *B. trifasciatus* for subsequent ensemble modeling. The ensemble model for *C. amoena* achieved AUC = 0.978 and TSS = 0.885, while that for *B. trifasciatus* achieved AUC = 0.958 and TSS = 0.807. Both ensemble models outperformed individual models, indicating higher predictive accuracy and reliability.

### 3.2. Contribution of Environmental Variables

In species distribution modeling, we quantified the influence of each predictor by assessing the percentage contribution of environmental variables to the predicted distribution patterns [62]. According to the modeling analysis results, the contribution rates of three major categories of environmental variables—climate, topography, and anthropogenic influence—to the distribution of *C. amoena* and *B. trifasciatus* are as follows: climate variables (*C. amoena* 84.96%, *B. trifasciatus* 71.76%), topographic variables (*C. amoena* 11.92%, *B. trifasciatus* 23.97%), and anthropogenic variables (*C. amoena* 3.12%, *B. trifasciatus* 4.27%). This indicates that climate variables are the dominant factors influencing the potential distribution patterns of both species.

The key environmental factor for *C. amoena* was annual precipitation (Bio12, 40.92%), while for *B. trifasciatus* it was precipitation of the driest quarter (Bio17, 40.23%). Both variables are precipitation-related, which suggests that precipitation-related predictors are the primary climatic determinants in the ensemble models (Table 3).

### 3.3. Current Potential Suitable Distribution of C. amoena and Its Pollinator

**Figure 2:**
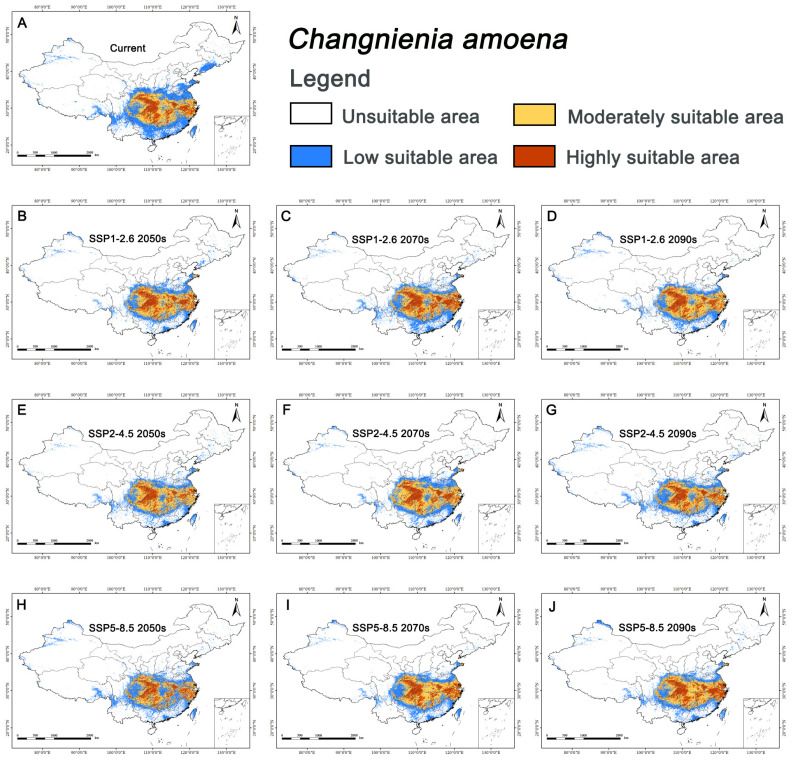
Habitat suitability of *Changnienia amoena* in China under different climate scenarios. (**A**) Suitable area for *C. amoena* under current scenario; (**B**–**D**) Its suitable areas under SSP1-2.6 scenario in 2050s, 2070s, and 2090s, respectively; (**E**–**G**) Its suitable areas under SSP2-4.5 scenario in 2050s, 2070s, and 2090s, respectively; (**H**–**J**) Its suitable areas under SSP5-8.5 scenario in 2050s, 2070s, and 2090s, respectively.

Under current climate conditions, the suitable habitat area (medium and high suitability) for *C. amoena* was $108.06\times 10^{4}\;{\mathrm{km}}^{2}$, accounting for 11.26% of China’s land area; the highly suitable area was $39.25\times 10^{4}\;{\mathrm{km}}^{2}$, accounting for 4.09% of China’s land area (Table 4). Under the current climatic scenario, the potentially suitable habitats for *C*. *amoena* are mainly concentrated in southern Jiangsu, southern Anhui, northern Jiangxi, and northern Zhejiang in eastern China; southern Henan, central Hunan, and Hubei in central China; and Chongqing and Sichuan in southwestern China, with scattered distributions in southern Shaanxi (Figure 2A).

**Figure 3:**
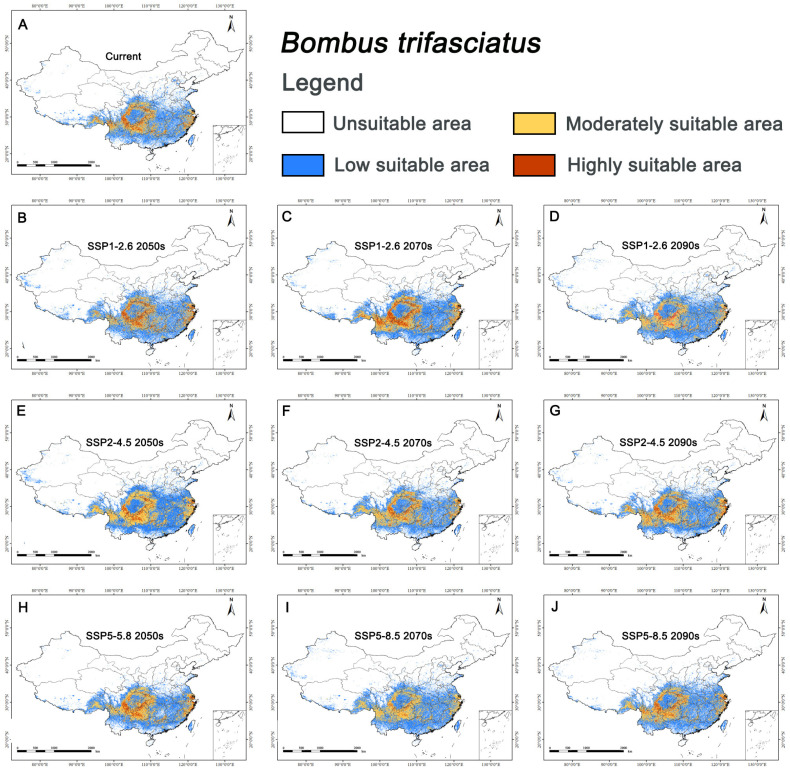
Habitat suitability of *Bombus trifasciatus* in China under different climate scenarios. (**A**) Suitable area for *B. trifasciatus* under current scenario; (**B**–**D**) Its suitable areas under SSP1-2.6 scenario in 2050s, 2070s, and 2090s, respectively; (**E**–**G**) Its suitable areas under SSP2-4.5 scenario in 2050s, 2070s, and 2090s, respectively; (**H**–**J**) Its suitable areas under SSP5-8.5 scenario in 2050s, 2070s, and 2090s, respectively.

For *B. trifasciatus*, the suitable habitat area (low, medium, and high suitability) was $232.02\times 10^{4}\;{\mathrm{km}}^{2}$, accounting for 24.17% of China’s land area; and the highly suitable area was $15.95\times 10^{4}\;{\mathrm{km}}^{2}$, accounting for 1.66% of China’s land area. Its potential distribution was primarily in Zhejiang, northern Fujian, southern Anhui in eastern China, western Hubei in central China, southern Sichuan, Guizhou, northern Yunnan, southeastern Tibet, and central Chongqing in southwestern China, with scattered occurrences in southern Shaanxi, southern Inner Mongolia, and Guangxi (Figure 3A).

Overall, under the current climate, the endangered *C. amoena* has a relatively restricted suitable habitat confined to central-eastern and some southwestern mountainous regions. In contrast, its pollinator *B. trifasciatus* has a much wider distribution, indicating spatial asymmetry between the two.

### 3.4. Future Distribution of C. amoena and Its Pollinator

For *C. amoena*, suitable habitat area (medium and high suitability) decreased in most future scenarios except under SSP2-4.5 in 2061–2080 and SSP5-8.5 in 2081–2100 (Table 4, Figure 2B–J). The mean future suitable area was $103.84\times 10^{4}\;{\mathrm{km}}^{2}$ (10.82% of China’s land area), lower than the current area. The maximum area ($111.34\times 10^{4}\;{\mathrm{km}}^{2}$) occurred under SSP5-8.5 in 2081–2100, while the minimum ($92.43\times 10^{4}\;{\mathrm{km}}^{2}$) occurred under SSP5-8.5 in 2041–2060.

For *B. trifasciatus*, suitable habitat area (low, medium, and high suitability) increased in most future scenarios except under SSP1-2.6 in 2081–2100 and SSP5-8.5 in 2041–2060. The mean future suitable area was $243.20\times 10^{4}\;{\mathrm{km}}^{2}$ (25.33%), higher than the current area. The maximum area ($263.29\times 10^{4}\;{\mathrm{km}}^{2}$) occurred under SSP2-4.5 in 2041–2060, and the minimum ($220.17\times 10^{4}\;{\mathrm{km}}^{2}$) under SSP1-2.6 in 2081–2100 (Figure 3B–J).

**Figure 4:**
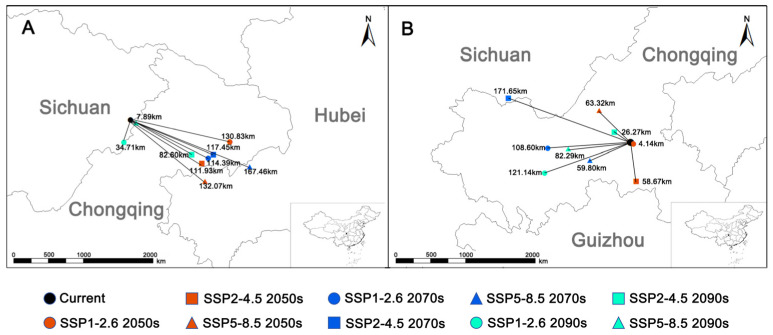
Centroid shift in the *Changnienia amoena* and its pollinator in a suitable area under the current and future different climate scenarios. (**A**): *Changnienia amoena*; (**B**): *Bombus trifasciatus*.

Centroid shifts revealed a spatial mismatch between *C. amoena* and its pollinator. For *C. amoena*, centroids shifted mainly southeastward, with an average displacement of 99.93 km; the maximum shift (167.46 km) occurred under SSP5-8.5 in 2061–2080, and the minimum (7.89 km) under SSP5-8.5 in 2081–2100. Only under SSP1-2.6 in 2081–2100 did the centroid shift southwest. For *B. trifasciatus*, centroids shifted mainly westward, with an average displacement of 77.32 km; the maximum shift (171.65 km) occurred under SSP2-4.5 in 2061–2080, and the minimum (4.14 km) under SSP1-2.6 in 2041–2060. Only under SSP1-2.6 and SSP2-4.5 in 2041–2060 did centroids shift southeast (Figure 4).

### 3.5. Niche Overlap Between C. amoena and Its Pollinator

Under current climatic conditions, Schoener’s *D* between *C. amoena* and its pollinator was 0.712 (Table 5), indicating high niche overlap (0.60–0.80) and broadly similar environmental requirements. Across the nine future climate scenarios, the *D* values for this species pair showed a decreasing trend. The future mean value (*D* = 0.672) was lower than the current value (*D* = 0.712), suggesting a slight reduction in niche overlap between the two species. Meanwhile, the trend in Hellinger’s *I* values was broadly consistent with that of the *D* values. The future mean value (*I* = 0.896) was lower than the current value (*I* = 0.899), further supporting the likelihood of a slight weakening in niche overlap under future climate conditions.

## 4. Discussion

### 4.1. Ensemble Model Assessment and Key Environmental Factors

This study employed the Biomod2 platform to predict the potential distributions of *C. amoena* and its obligate pollinator. For this orchid, its performance metrics for the ensemble model (AUC = 0.978, TSS = 0.885) demonstrate a high level of predictive accuracy and reliability (Table 2). Consistent with the findings of Xu et al. [63] for the endangered orchid *Cypripedium japonicum*, our results reinforce that the ensemble model with multiple algorithms can yield more accurate predictions than a single model. Likewise, for its pollinator, the performance of the ensemble model (AUC = 0.958, TSS = 0.807) is also generally better than that of an individual model.

Our projections indicate that climatic variables are the dominant factors governing the geographical distribution pattern of *C. amoena* relative to topography and anthropogenic influence. The top three key environmental predictors are annual precipitation (Bio12), minimum temperature of the coldest month (Bio06), and temperature seasonality (Bio04). Evidently, Bio12 accounted for a dominant contribution of 40.92% among the nine environmental variables considered. This prominent role strongly corresponds to the species’ ecological habit as an understory perennial herb favoring shady and humid conditions [24], confirming that water availability is the primary constraint on its potential distribution. Similarly, the distribution of its pollinator *B. trifasciatus* is primarily driven by climatic factors. The top three key predictors are precipitation of the driest quarter (Bio17), minimum temperature of the coldest month (Bio06), and slope. The high contribution of Bio17 (40.23%) suggests that water availability during seasonal droughts is crucial because it may potentially affect larval development, adult activity, and the availability of nectar sources. Notably, temperature and topography are identified as the common factors between them, indicating that both exerted subsidiary influence on the distribution of these species. Additionally, anthropogenic factors showed limited effects. This is likely because they are mainly distributed in subtropical mountainous areas, where it is relatively difficult for humans to access, or some parts are located within nature reserves [23]. Therefore, the precipitation-related climate is considered the most crucial factor shaping the distribution of this orchid and its pollinators.

Our results differ from those of others [22,32], which highlighted the substantial influence of temperature-related variables on the orchid distribution. Such a discrepancy can be mainly attributed to differences in sample representativeness. For example, the occurrence records of *C. amoena* in this study (i.e., 123 records) span a greater number of China’s provinces than those in Liu et al. [22] (only 69 records).

### 4.2. Suitable Distribution and Future Changes for C. amoena and Its Pollinator

Model projections indicate that the current potentially suitable habitat for *C. amoena* is primarily located in eastern China (Anhui, Jiangsu, Jiangxi, and Zhejiang), central China (Henan, Hubei and Hunan), and southwestern China (Chongqing and Sichuan). The total suitable area of *C. amoena* within China is estimated at 108.06 × 10^4^ km^2^, accounting for approximately 11.26% of the country’s total land area. This area is substantially smaller than the result reported by Liu et al. [22] (43.56 × 10^5^ km^2^), but larger than that by Liu et al. [32] (16.47 × 10^4^ km^2^). Furthermore, our model projections showcase a contraction in the suitable habitat for *C. amoena*, with its distribution centroid shifting southeastward.

Compared with the orchid, its pollinator exhibited a larger suitable area, and its distribution centroid is anticipated to shift westward as its climatically suitable habitat changes. Therefore, these divergent trends in range dynamics and directional shifts under climate change imply that global warming may be particularly unfavorable for the distribution and persistence of the endangered orchid.

It is noteworthy that our results differ significantly from previous studies regarding the suitable habitats and future changes in the orchid and its pollinators under both current and future climate scenarios. The main reasons are as follows:

(1) Model algorithm: Ensemble modeling can generate more reliable projections than single-algorithm methods [34]. In this study, we used the Biomod2 platform to construct ensemble models for each species, thus improving prediction reliability. In contrast, Liu et al. [22] used only MaxEnt. Although they further applied Geographically and Temporally Weighted Regression (GTWR), we contend that this approach does not fully leverage the complementary strengths of multiple modeling algorithms.
(2) Sampling representativeness: After spatial rarefaction, our analysis used 123 occurrence points for *C. amoena* and 43 for *B. trifasciatus*, ensuring better data coverage. By contrast, Liu et al. [22] included only 69 and 34 records for the two species, and Liu et al. [32] used merely 48 points for *C. amoena*, representing relatively limited samples. Take the occurrence points of the orchid as an example. Our dataset added more distribution records from northwestern, southern, and central China, which significantly improved the spatial representativeness of the samples. Such geographical sampling bias can lead to sampling selection bias, potentially causing models to overestimate suitability in sampled regions and underestimate potential in unsampled areas [33].
(3) Environmental variables: There are considerable differences in the selection of environmental variables for *C. amoena* when modeling. Some variables have weak ecological relevance to their distribution and cannot effectively reflect their habitat requirements. For example, Liu et al. [22] included wind speed in MaxEnt modeling. However, as a typical understory herb, *C. amoena* lives in microhabitats where wind effects are strongly moderated by the forest canopy, which raises doubts about the ecological rationality of including wind-related variables in its modeling.
(4) Habitat suitability classification: The MaxSSS method applied in our study, recognized for its high sensitivity, has been widely adopted in modeling endangered plant species [35]. In contrast, the natural breaks method used in previous studies may fail to optimally balance prediction deviations, potentially resulting in bias in suitable range estimation [51].

### 4.3. Declining Niche Overlap and Its Implications for Obligate Pollination Systems

The niche overlap index reflects the similarity in environmental preferences among species and serves as a metric for assessing their coexistence potential [64]. Our projections indicate a declining trend in future niche overlap between *C. amoena* and *B. trifasciatus*. This pollinating insect is currently recognized as the sole effective and specialized pollinator of *C. amoena* in the wild, with its proboscis structure precisely adapted to the floral morphology involved in the orchid’s deceptive pollination strategy [27]. Pollination is essential for sexual reproduction and can constrain a plant’s geographical range [65]. For orchids, specialized pollination mechanisms may develop distinct morphological features tailored to specific insect pollinators, potentially enhancing genetic diversity and seed production, thereby contributing to the long-term viability of plant populations [66]. As a result, obligate pollination systems are critically dependent on a high degree of spatiotemporal synchronization between the interacting species, resulting in a more pronounced interdependence. Consequently, despite a potential expansion in the pollinator’s suitable range, the decreasing niche overlap indicates a reduction in spatial congruence between the two species. This mismatch may lower pollination efficiency, which could further compromise the orchid’s naturally low rates of fruit set and sexual reproduction, intensifying its overall endangerment.

### 4.4. Conservation Implications for C. amoena

This study employed an ensemble modeling approach to systematically evaluate the potentially suitable distributions of *C. amoena* and its pollinator. Our analysis revealed that the suitable habitat for *C. amoena* covers approximately 1.0806 million km^2^, representing 11.26% of China’s land area, primarily in the central, eastern, and southern regions of this country. Our results suggest that *C. amoena* is subject to severe pressure from both projected habitat contraction and increasing mismatch with its obligate pollinator under future climate scenarios. Integrating the biological characteristics of *C. amoena*, we propose the following conservation recommendations:

The first priority concerns the conservation coverage for this endangered orchid. Although the orchid is listed as a second-level nationally protected wild plant in China, currently only a small portion of its current distribution is located within national or provincial nature reserves. Accordingly, future conservation efforts should consider establishing a natural mini-reserve or appropriately expanding the boundaries of existing protected areas. Moreover, conservation planning within nature reserves must explicitly include the synergistic protection of both *C. amoena* and its specific pollinator *B. trifasciatus*. In addition, priority should be given to safeguarding key areas that currently exhibit high niche overlap and are projected to remain suitable under future climates, especially those habitats with favorable moisture conditions.

The second priority concerns the genetic sampling of the orchid. Lu et al. [67] utilized genome skimming to annotate and compare the complete plastomes of *C. amoena* sampled from seven provinces across China, revealing low overall plastome variation within the species. Li and Ge [25] employed the RAPD technique to assess genetic diversity among 216 individuals sampled from 11 populations across five provinces. Their results indicated that the species maintains intermediate levels of genetic diversity at the species level yet exhibits significantly low diversity within populations, alongside an exceptionally high level of population differentiation. Our projection suggests that the current suitable habitat range of *C. amoena* encompasses more than 12 provinces in China. Therefore, systematic sampling should be undertaken to fully capture the species’ genetic diversity and structure across its entire distribution, thereby providing a scientific basis for developing informed ex situ conservation and genetic management strategies.

Furthermore, future research should extend its scope to encompass not only the interactive relationships between the orchid and its pollinator but also the symbiotic mechanisms involving mycorrhizal fungi. A comprehensive understanding of these dual biotic interactions contributes to formulating more targeted and effective conservation strategies for this species.

## 5. Conclusions

This study, for the first time, reveals contrasting responses of the endangered orchid *C. amoena* and its obligate pollinator *B. trifasciatus* to climate change through the Biomod2 platform in China. While the pollinator’s suitable habitat is projected to expand westward, the orchid’s climatically suitable habitat may contract slightly and shift southeastward, leading to a potential decrease in their niche overlap. These divergent distributional shifts underscore the risk of spatial decoupling in this highly specialized mutualism under future climates. Our findings, derived from robust ensemble modeling, contribute to understanding climate impact on species interactions and highlight the necessity of incorporating pollinator dynamics into orchid conservation planning. A limitation of this study is the omission of biotic interactions such as mycorrhizal associations and soil properties, which could refine future distribution models.

## Figures and Tables

**Table 1:** Description of 23 environmental variables of the endangered *Changnienia amoena* and its pollinator *Bombus trifasciatus* in China.

Category	Variable	Description	Unit
Bioclimate	Bio1	Annual mean temperature	°C
	Bio2	Mean diurnal range (mean of monthly (max temp–min temp))	°C
	Bio3	Isothermality ((Bio2/Bio7) × 100)	%
	Bio4	Temperature seasonality (standard deviation × 100)	-
	Bio5	Max temperature of warmest month	°C
	Bio6	Min temperature of coldest month	°C
	Bio7	Temperature annual range (Bio5–Bio6)	°C
	Bio8	Mean temperature of wettest quarter	°C
	Bio9	Mean temperature of driest quarter	°C
	Bio10	Mean temperature of warmest quarter	°C
	Bio11	Mean temperature of coldest quarter	°C
	Bio12	Annual precipitation	mm
	Bio13	Precipitation of wettest month	mm
	Bio14	Precipitation of driest month	mm
	Bio15	Precipitation seasonality (coefficient of variation)	-
	Bio16	Precipitation of wettest quarter	mm
	Bio17	Precipitation of driest quarter	mm
	Bio18	Precipitation of warmest quarter	mm
	Bio19	Precipitation of coldest quarter	mm
Terrain	Elevation	-	m
	Slope	-	°
	Aspect	-	°
Anthropogenic factor	HI	Human influence	-

**Table 2:** Mean area under the curve (AUC) and true skill statistic (TSS) values for each modeling algorithm and integrated model (bold) for the endangered *Changnienia amoena* and its pollinator *Bombus trifasciatus* under the current climate scenario.

	Orchid: *Changnienia amoena*		Pollinator: *Bombus trifasciatus*	
Model	AUC	TSS	AUC	TSS
ANN	0.765	0.530	0.678	0.344
CTA	0.944	0.865	0.842	0.672
FDA	0.922	0.777	0.895	0.680
GAM	0.926	0.783	0.902	0.713
GBM	0.981	0.905	0.972	0.851
GLM	0.978	0.914	0.938	0.782
MARS	0.976	0.901	0.963	0.842
MAXENT	0.975	0.879	0.920	0.754
RF	1.000	1.000	1.000	1.000
SRE	0.734	0.468	0.657	0.314
**Ensemble model**	**0.978**	**0.885**	**0.958**	**0.807**

**Table 3:** Four key environmental variables influencing the potential distribution of the endangered *Changnienia amoena* and its pollinator *Bombus trifasciatus*.

Species	No.	Variable	Percent Contribution (%)
Orchid: *Changnienia amoena*	1	Bio12	40.92
	2	Bio06	26.61
	3	Bio04	13.52
	4	Slope	10.28
Pollinator: *Bombus trifasciatus*	1	Bio17	40.23
	2	Bio06	15.85
	3	Slope	14.70
	4	Bio07	11.01

**Table 4:** Projected changes in suitable habitat area for the endangered *Changnienia amoena* and its pollinator *Bombus trifasciatus* under different climate scenarios.

Scenarios	Low Suitable Area		Moderately Suitable Area		Highly Suitable Area		Suitable Area	
	Area	Trend	Area	Trend	Area	Trend	Area	Trend
	(×10^4^ km^2^)	(%)	(×10^4^ km^2^)	(%)	(×10^4^ km^2^)	(%)	(×10^4^ km^2^)	(%)
*Changnienia amoena*								
Current	132.22	-	68.81	-	39.26	-	108.06	-
SSP1-2.6								
2041–2060	78.50	↓40.63	66.00	↓4.09	40.57	↑3.36	106.57	↓1.38
2061–2080	88.79	↓32.85	62.54	↓9.10	43.43	↑10.64	105.97	↓1.93
2081–2100	77.33	↓41.52	68.23	↓0.83	38.57	↓1.73	106.81	↓1.16
Average	81.54	↓38.33	65.59	↓4.67	40.86	↑4.09	106.45	↓1.49
SSP2-4.5								
2041–2060	79.77	↓39.67	69.19	↑0.55	33.02	↓15.87	102.21	↓5.42
2061–2080	79.31	↓40.02	72.72	↑5.68	38.11	↓2.91	110.83	↑2.56
2081–2100	91.59	↓30.73	62.27	↓9.50	34.82	↓11.29	97.10	↓10.15
Average	83.56	↓36.80	68.06	↓1.09	35.32	↓10.02	103.38	↓4.34
SSP5-8.5								
2041–2060	98.34	↓25.62	59.81	↓13.08	32.63	↓16.89	92.43	↓14.46
2061–2080	80.59	↓39.05	63.04	↓8.39	38.23	↓2.60	101.27	↓6.28
2081–2100	78.72	↓40.46	67.01	↓2.61	44.33	↑12.93	111.34	↑3.04
Average	85.88	↓35.04	63.29	↓8.03	38.40	↓2.18	101.68	↓5.90
Mean future climate scenario value	83.66	↓36.73	65.65	↓4.60	38.19	↓2.71	103.84	↓3.91
*Bombus trifasciatus*								
Current	143.16	-	72.92	-	15.95	-	232.02	-
SSP1-2.6								
2041–2060	148.40	↑3.66	75.62	↑3.70	20.41	↑27.99	244.43	↑5.35
2061–2080	150.95	↑5.44	78.85	↑8.14	25.89	↑62.35	255.69	↑10.20
2081–2100	132.08	↓7.74	70.40	↓3.46	17.69	↑10.94	220.17	↓5.11
Average	143.81	↑0.45	74.96	↑2.80	21.33	↑33.76	240.10	↑3.48
SSP2-4.5								
2041–2060	156.86	↑9.57	92.82	↑27.30	13.62	↓14.60	263.29	↑13.48
2061–2080	138.83	↓3.02	79.22	↑8.64	14.19	↓11.00	232.24	↑0.09
2081–2100	143.30	↑0.10	81.60	↑11.91	17.73	↑11.20	242.63	↑4.57
Average	146.33	↑2.21	84.55	↑15.95	15.18	↓4.80	246.05	↑6.05
SSP5-8.5								
2041–2060	132.31	↓7.58	79.43	↑8.93	17.33	↑8.70	229.07	↓1.27
2061–2080	161.89	↑13.08	84.58	↑15.99	6.63	↓58.43	253.10	↑9.08
2081–2100	151.28	↑5.67	80.96	↑11.04	15.91	↓0.20	248.16	↑6.95
Average	148.49	↑3.73	81.66	↑11.99	13.29	↓16.64	243.44	↑4.92
Mean future climate scenario value	146.21	↑2.13	80.39	↑10.24	16.60	↑4.11	243.20	↑11.17

Note: “Suitable area” is defined differently for the two species: for *Changnienia amoena*, suitable area = medium + high; for *Bombus trifasciatus*, suitable area = low + medium + high. Up arrow (↑) means increase compared to the current; down arrow (↓) means decrease.

**Table 5:** Niche overlap in terms of Schoener’s parameter (*D*) and Hellinger’s parameter (*I*) of the endangered *Changnienia amoena* and its pollinator, *Bombus trifasciatus*, under different climate scenarios.

Climate Scenarios		*Changnienia amoena* vs. *Bombus trifasciatus*	
		*D*	*I*
Current		0.712	0.899
2041–2060	SSP1-2.6	0.660↓	0.885↓
	SSP2-4.5	0.688↓	0.909↑
	SSP5-8.5	0.711↓	0.916↑
2061–2080	SSP1-2.6	0.661↓	0.889↓
	SSP2-4.5	0.659↓	0.884↓
	SSP5-8.5	0.652↓	0.887↓
2081–2100	SSP1-2.6	0.658↓	0.884↓
	SSP2-4.5	0.683↓	0.906↑
	SSP5-8.5	0.674↓	0.900↑
Mean ± SD		0.672 ± 0.019	0.896 ± 0.012

Note: Mean ± SD refers to the average value of Schoener’s *D* and Hellinger’s *I* of each species pair under nine future climate scenarios. Up arrow (↑) means increase compared to the current; down arrow (↓) means decrease.

## Data Availability

Data are contained within the article and Supplementary Materials.

## Supplementary Materials

The following supporting information can be downloaded at: https://www.mdpi.com/article/10.3390/biology15060485/s1, Table S1: Occurrence records of the endangered *Changnienia amoena* and its pollinator *Bombus trifasciatus* in China.
