# Supplementary material for: Contrasting Range Shifts of an Endangered Orchid Changnienia amoena and Its Obligate Pollinator Under Climate Change in China
Source: Biology (Basel). 2026 Mar 19;15(6):485. doi: 10.3390/biology15060485 (PMC13023849; doi:10.3390/biology15060485)
Supplement: Supplementary file 1 [file biology-15-00485-s001.zip › biology-4184230-supplementary.pdf]

Supplementary Table S1 Occurrence records of the endangered *Changnienia amoena* and its pollinator *Bombus trifasciatus* in China.

Species	North longitude (°)	East latitude (°)
Orchid: *Changnienia amoena*	103.60	31.40
Orchid: *Changnienia amoena*	104.82	32.59
Orchid: *Changnienia amoena*	104.96	32.02
Orchid: *Changnienia amoena*	105.25	32.76
Orchid: *Changnienia amoena*	105.74	33.02
Orchid: *Changnienia amoena*	105.92	33.10
Orchid: *Changnienia amoena*	106.15	32.74
Orchid: *Changnienia amoena*	106.38	32.79
Orchid: *Changnienia amoena*	106.44	32.85
Orchid: *Changnienia amoena*	106.81	30.30
Orchid: *Changnienia amoena*	108.67	27.53
Orchid: *Changnienia amoena*	109.12	30.04
Orchid: *Changnienia amoena*	109.27	32.09
Orchid: *Changnienia amoena*	110.08	29.71
Orchid: *Changnienia amoena*	110.68	30.01
Orchid: *Changnienia amoena*	110.72	26.50
Orchid: *Changnienia amoena*	110.92	26.32
Orchid: *Changnienia amoena*	111.07	26.65
Orchid: *Changnienia amoena*	111.19	26.54
Orchid: *Changnienia amoena*	112.00	34.10
Orchid: *Changnienia amoena*	112.72	27.30
Orchid: *Changnienia amoena*	113.29	32.38
Orchid: *Changnienia amoena*	113.95	31.69
Orchid: *Changnienia amoena*	114.08	31.82
Orchid: *Changnienia amoena*	114.27	31.63
Orchid: *Changnienia amoena*	115.79	31.14
Orchid: *Changnienia amoena*	115.97	29.52
Orchid: *Changnienia amoena*	117.48	30.04
Orchid: *Changnienia amoena*	119.09	32.13
Orchid: *Changnienia amoena*	119.45	31.43
Orchid: *Changnienia amoena*	119.48	30.36
Orchid: *Changnienia amoena*	121.09	29.73
Orchid: *Changnienia amoena*	116.02	31.19
Orchid: *Changnienia amoena*	117.50	30.10
Orchid: *Changnienia amoena*	116.31	31.36
Orchid: *Changnienia amoena*	115.92	31.69
Orchid: *Changnienia amoena*	105.74	33.03
Orchid: *Changnienia amoena*	105.50	32.94
Orchid: *Changnienia amoena*	108.70	27.92
Orchid: *Changnienia amoena*	114.04	32.13

Orchid: *Changnienia amoena*	110.01	30.45
Orchid: *Changnienia amoena*	109.82	30.19
Orchid: *Changnienia amoena*	110.37	31.44
Orchid: *Changnienia amoena*	115.91	31.01
Orchid: *Changnienia amoena*	114.61	31.30
Orchid: *Changnienia amoena*	114.66	31.47
Orchid: *Changnienia amoena*	116.01	31.00
Orchid: *Changnienia amoena*	115.68	30.75
Orchid: *Changnienia amoena*	115.81	31.10
Orchid: *Changnienia amoena*	110.27	31.47
Orchid: *Changnienia amoena*	110.10	31.46
Orchid: *Changnienia amoena*	110.54	30.74
Orchid: *Changnienia amoena*	109.86	29.00
Orchid: *Changnienia amoena*	110.17	29.42
Orchid: *Changnienia amoena*	110.54	29.13
Orchid: *Changnienia amoena*	119.09	32.14
Orchid: *Changnienia amoena*	118.91	30.11
Orchid: *Changnienia amoena*	119.44	30.37
Orchid: *Changnienia amoena*	109.88	31.08
Orchid: *Changnienia amoena*	104.05	30.65
Orchid: *Changnienia amoena*	110.39	31.44
Orchid: *Changnienia amoena*	116.04	31.41
Orchid: *Changnienia amoena*	119.46	30.34
Orchid: *Changnienia amoena*	110.08	31.29
Orchid: *Changnienia amoena*	118.20	29.38
Orchid: *Changnienia amoena*	114.63	31.54
Orchid: *Changnienia amoena*	104.82	31.99
Orchid: *Changnienia amoena*	116.03	30.99
Orchid: *Changnienia amoena*	105.49	33.00
Orchid: *Changnienia amoena*	109.45	30.28
Orchid: *Changnienia amoena*	110.98	30.83
Orchid: *Changnienia amoena*	111.33	30.76
Orchid: *Changnienia amoena*	113.43	32.38
Orchid: *Changnienia amoena*	101.50	27.42
Orchid: *Changnienia amoena*	109.87	31.11
Orchid: *Changnienia amoena*	109.36	27.57
Orchid: *Changnienia amoena*	113.01	28.21
Orchid: *Changnienia amoena*	118.80	32.06
Orchid: *Changnienia amoena*	112.73	27.24
Orchid: *Changnienia amoena*	115.97	29.34
Orchid: *Changnienia amoena*	115.99	29.67
Orchid: *Changnienia amoena*	119.17	31.92
Orchid: *Changnienia amoena*	119.44	30.35
Orchid: *Changnienia amoena*	110.26	26.49

Orchid: *Changnienia amoena*	116.00	29.59
Orchid: *Changnienia amoena*	110.08	31.22
Orchid: *Changnienia amoena*	109.00	29.60
Orchid: *Changnienia amoena*	115.92	29.49
Orchid: *Changnienia amoena*	115.80	31.14
Orchid: *Changnienia amoena*	115.78	31.17
Orchid: *Changnienia amoena*	107.09	29.18
Orchid: *Changnienia amoena*	119.32	31.79
Orchid: *Changnienia amoena*	115.96	29.46
Orchid: *Changnienia amoena*	103.95	31.84
Orchid: *Changnienia amoena*	116.02	29.52
Orchid: *Changnienia amoena*	115.93	29.52
Orchid: *Changnienia amoena*	110.68	31.75
Orchid: *Changnienia amoena*	109.50	29.49
Orchid: *Changnienia amoena*	110.45	34.46
Orchid: *Changnienia amoena*	110.61	29.05
Orchid: *Changnienia amoena*	110.87	26.41
Orchid: *Changnienia amoena*	111.11	26.34
Orchid: *Changnienia amoena*	116.58	31.09
Orchid: *Changnienia amoena*	105.23	32.68
Orchid: *Changnienia amoena*	110.47	25.94
Orchid: *Changnienia amoena*	108.62	27.55
Orchid: *Changnienia amoena*	108.87	27.77
Orchid: *Changnienia amoena*	114.07	31.82
Orchid: *Changnienia amoena*	111.93	33.51
Orchid: *Changnienia amoena*	110.50	31.33
Orchid: *Changnienia amoena*	110.89	31.60
Orchid: *Changnienia amoena*	110.03	31.21
Orchid: *Changnienia amoena*	115.86	31.10
Orchid: *Changnienia amoena*	115.85	30.98
Orchid: *Changnienia amoena*	111.13	26.71
Orchid: *Changnienia amoena*	109.36	32.03
Orchid: *Changnienia amoena*	106.46	32.82
Orchid: *Changnienia amoena*	106.26	32.83
Orchid: *Changnienia amoena*	106.01	33.03
Orchid: *Changnienia amoena*	109.53	31.95
Orchid: *Changnienia amoena*	108.36	31.95
Orchid: *Changnienia amoena*	114.92	28.87
Orchid: *Changnienia amoena*	114.15	26.64
Pollinator: *Bombus trifasciatus*	120.18	30.24
Pollinator: *Bombus trifasciatus*	95.15	29.12
Pollinator: *Bombus trifasciatus*	110.39	31.75
Pollinator: *Bombus trifasciatus*	122.11	30.03
Pollinator: *Bombus trifasciatus*	105.61	27.78

Pollinator: *Bombus trifasciatus*	103.34	29.54
Pollinator: *Bombus trifasciatus*	119.72	30.00
Pollinator: *Bombus trifasciatus*	108.84	23.87
Pollinator: *Bombus trifasciatus*	102.75	29.82
Pollinator: *Bombus trifasciatus*	108.70	27.84
Pollinator: *Bombus trifasciatus*	102.37	26.58
Pollinator: *Bombus trifasciatus*	120.96	28.80
Pollinator: *Bombus trifasciatus*	117.67	27.67
Pollinator: *Bombus trifasciatus*	117.40	27.50
Pollinator: *Bombus trifasciatus*	102.86	29.98
Pollinator: *Bombus trifasciatus*	102.43	27.48
Pollinator: *Bombus trifasciatus*	118.17	26.65
Pollinator: *Bombus trifasciatus*	108.41	30.81
Pollinator: *Bombus trifasciatus*	104.69	38.07
Pollinator: *Bombus trifasciatus*	107.71	34.09
Pollinator: *Bombus trifasciatus*	119.87	30.25
Pollinator: *Bombus trifasciatus*	107.34	26.25
Pollinator: *Bombus trifasciatus*	103.28	29.58
Pollinator: *Bombus trifasciatus*	119.48	30.39
Pollinator: *Bombus trifasciatus*	103.38	29.58
Pollinator: *Bombus trifasciatus*	111.91	24.78
Pollinator: *Bombus trifasciatus*	121.78	29.80
Pollinator: *Bombus trifasciatus*	110.05	31.35
Pollinator: *Bombus trifasciatus*	120.15	30.27
Pollinator: *Bombus trifasciatus*	99.64	27.90
Pollinator: *Bombus trifasciatus*	102.73	30.27
Pollinator: *Bombus trifasciatus*	102.77	30.25
Pollinator: *Bombus trifasciatus*	118.40	31.05
Pollinator: *Bombus trifasciatus*	107.78	33.60
Pollinator: *Bombus trifasciatus*	100.04	27.50
Pollinator: *Bombus trifasciatus*	99.03	27.67
Pollinator: *Bombus trifasciatus*	98.91	28.49
Pollinator: *Bombus trifasciatus*	99.82	28.57
Pollinator: *Bombus trifasciatus*	99.25	30.54
Pollinator: *Bombus trifasciatus*	103.36	29.55
Pollinator: *Bombus trifasciatus*	102.83	30.26
Pollinator: *Bombus trifasciatus*	99.64	27.91
Pollinator: *Bombus trifasciatus*	106.47	30.75
